# Solubility and ADMET profiles of short oligomers of lactic acid

**DOI:** 10.5599/admet.843

**Published:** 2020-06-28

**Authors:** Daniela Dascălu, Diana Larisa Roman, Madalina Filip, Alecu Ciorsac, Vasile Ostafe, Adriana Isvoran

**Affiliations:** 1 Department of Biology-Chemistry and Advanced Environmental Research Laboratories, West University of Timișoara, Timișoara, Romania; 2 Department of Physical Education and Sport, University Politehnica Timișoara, Timișoara, Romania

**Keywords:** Pharmacokinetics, toxicological endpoints, biodegradation products of PLA

## Abstract

Polylactic acid (PLA) is a polymer with an increased potential to be used in different medical applications, including tissue engineering and drug-carries. The use of PLA in medical applications implies the evaluation of the human organism's response to the polymer inserting and to its degradation products. Consequently, within this study, we have investigated the solubility and ADMET profiles of the short oligomers (having the molecular weight lower than 3000 Da) resulting in degradation products of PLA. There is a linear decrease of the molar solubility of investigated oligomers with molecular weight. The results that are obtained also reveal that short oligomers of PLA have promising pharmacological profiles and limited toxicological effects on humans. These oligomers are predicted as potential inhibitors of the organic anion transporting peptides OATP1B1 and OATP1B3, they present minor probability to affect the androgen and glucocorticoid receptors, have a weak potential of hepatotoxicity, and may produce eye injuries. These outcomes may be used to guide or to supplement in vitro and/or in vivo toxicity tests such as to enhance the biodegradation properties of the biopolymer.

## Introduction

For a few decades, biopolymers are extensively in use as food additives, cosmetics, medical materials, water treatment chemicals, packaging, etc. Because of their intrinsic properties, especially non-cytotoxicity, biocompatibility and biodegradation, biopolymers are a class of materials that provides a wide range of applications in medicine [1]. They are considered outstanding candidates to be used for the preparation of many medical and body implants and also as controlled drug delivery systems.

**Figure 1.**
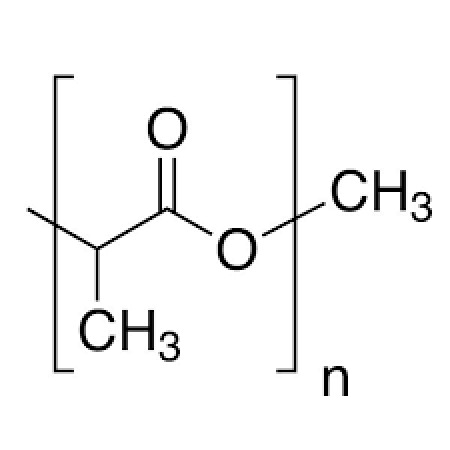
The structural formula of poly-lactic acid

Polylactic acid (PLA), with the chemical formula (C_3_H_4_O_2_)_n_ and illustrated in Figure 1, is one of the most commercially competitive polymers used for medical applications acting as biologically inert supporting materials as scaffolds or drug-carriers [2, 3]. PLA is bio absorbable being transformed in the human organism by simple hydrolysis to products that can be further metabolized or excreted [4, 5]. The biomedical applications of the polylactic acid (PLA) are drug delivery systems, suture threads, bone fixation screws [3].

PLA is degraded *in situ* through hydrolysis with the production of oligomers which are more water-soluble. Depending on the extent of hydrolysis, the products can be short oligomers (OLAs) and lactic acid (2-hydroxypropanoic acid the monomer) [3, 5]. Lactic acid is a non-toxic compound and a biochemical intermediate in carbohydrate metabolism [6]. The oligomers produced from hydrolysis may act as catalysts and they can diffuse from the sample toward the surfaces, these phenomena influencing the polymer degradation [3]. The counter-diffusion of the larger oligomers can be the determining phase for biodegradation as the oligomers having large dimensions cannot diffuse fast enough. Increased solubility of oligomers may facilitate diffusion and consequently, the biodegradability of the polymer.

It is already known that the degradation of biopolymers is important for controlled drug delivery systems and for various types of implants. Biopolymers can be degraded by random chain scission to oligomers with lower molecular weight [7]. The degradation products, the oligomers, may also affect the human organism, the effects they produce being dependent on absorption, distribution, metabolism, excretion and toxicity (ADMET) profile [8]. The solubility in aqueous media is one of the most important physicochemical characteristics influencing the ADMET properties [9, 10]. The soluble degradation products are either metabolized or transported through the lymphatic system to the kidney to be excreted from the organism [11].

PLA is known as a biodegradable, biocompatible, non-toxic and eco-friendly polymer [12] but there is a recent *in vitro* study revealing some toxic effects of PLA [13]: PLA inhibited bioluminescence (an endpoint illustrating cytotoxicity in mammalian cells) with high efficiency, induced an oxidative stress response and it contains estrogenic compounds. To the best of our knowledge, there are no available studies concerning the biological effects of OLAs. Taking into account this information, the aim of this study is to predict the aqueous solubility and ADMET profiles for short oligomers (containing up to 40 monomeric units and having the molecular weight up to 3000 Da) resulted from the degradation of PLA. Our methodology is based on the fact that controlling agencies in the field of drug and medical devices development have confidence in the results obtained by the use of computational tools for the prediction of the biological effects.

## Method

Within this study, we have considered short PLA oligomers (OLAs) containing from 1 to 40 lactic acid units. We used the SwissADME computational facility [14] to predict the values of their decimal logarithm of the molar solubility in water (log S) and their ADMET properties. Three methods are included in SwissADME to predict aqueous solubility: the *E*stimate *SOL*ubility (ESOL) model [15], a method adapted from Ali and co-workers [16], and a method based on a system of 16 fragmental contributions modulated by the squared root of molecular weight implemented under FILTER-IT software, version 1.0.2 (http://silicos-it.be.s3-website-eu-west-1.amazonaws.com/software/filter-it/1.0.2/filter-it.html). ESOL is a method used for estimating the aqueous solubility starting from its molecular topology [15] and is based on 4 molecular descriptors: the computed partition coefficient (clogP), the molecular weight (*M*_W_), the number of rotatable bonds (RB) and the proportion of heavy atoms in aromatic systems (AP) [15]. The equation allowing the computation of logS taking into account these descriptors is:


(1)

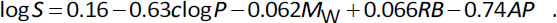



The model introduced by Ali and co-workers is based on two molecular descriptors: log *P* and topological polar surface area (TPSA) [16]:


(2)

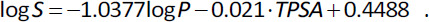



Besides the contribution of the other physicochemical parameters, these equations consider a linear decrease of the logarithm of the molar solubility coefficient with *M*_W_ and log *P*, respectively.

The calculation of the log *S* in Filter-IT software is based on the equation:


(3)

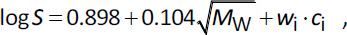



where *w*_i_ and *c*_i_ are the respective weights and counts for the fragment i, and *M*_W_ is the molecular weight of the compound (http://silicos-it.be.s3-website-eu-west-1.amazonaws.com/software/filter-it/1.0.2/filter-it.html).

SwissADME computational tool uses for lipophilicity descriptor (log *P*) in equations (1) and (2) the value computed using XLOGP3 method [17]. The input for the SwissADME tool is the structure of a chemical compound in the simplified molecular-input line-entry system (SMILES) format.

ADMET profiles, organ and genomic toxicity of OLAs have been predicted using admetSAR2.0 [18, 19], Pred-h-ERG, Pred-skin [20, 21], Endocrine Disruptome [22], Toxtree [23] and Carcino-PredEL [24]. We have selected these computational tools among the numerous available facilities, as they have the accuracy of prediction usually higher than 70% and friendly interfaces and tutorials that are available for free (online or open-source). A short description of the considered computational tools is given in Table 1. These computational tools have been used for assessing the ADMET profiles and toxicological endpoints for numerous classes of chemicals: chito-oligomers [8], synthetic steroids [25], cosmetic ingredients [26, 27], pesticides [26, 28], water-soluble derivatives of chitosan [29]. It demonstrates their wide-ranging applicability.

All the considered computational tools use as inputs SMILES (Simplified Molecular-Input Line-Entry System) formulas of the oligomers under investigation. These formulas have been obtained using ACD/ChemSketch utility (https://chemicalize.com accessed – accessed in March 2019). As it may be noticed from Table 1, none of these methods considers the concentration of the investigated compound when making predictions. This is one of the limitations of computational assessment of biological effects of chemicals based on expert rules and/or QSAR methods.

For scientific graphing and data analysis we have used Origin 8.0 software.

## Results and Discussions

**Figure 2.**
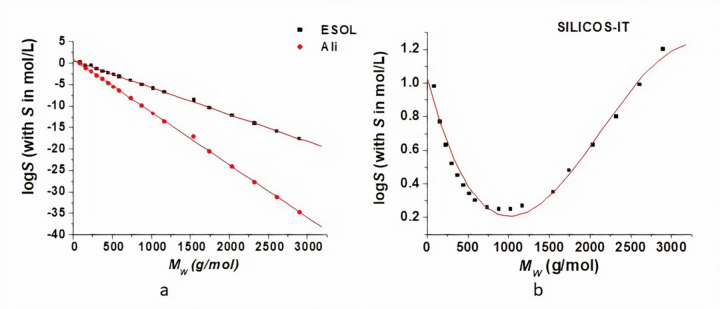
Solubility in water dependence on the molecular weight of the small oligomers of PLA: log *S* values are computed using ESOL and Ali methods (a) and SILICOS-IT method (b). Red lines correspond to the fitting of data, linear fit for the values obtained using ESOL and Ali methods (a) and polynomial fit of order 3 for the values obtained using SILICOS-IT method (b).

**Figure 3.**
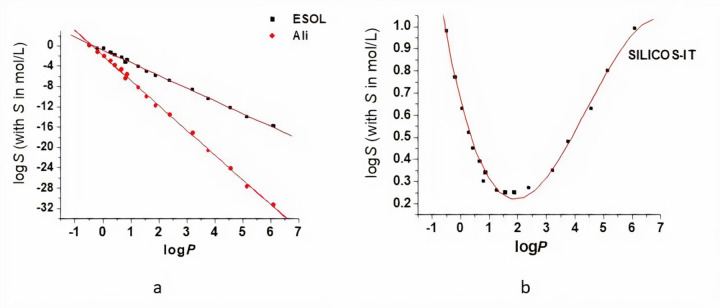
Solubility in water dependence on the log *P* values for the small oligomers of PLA: log *S* values are computed using ESOL and Ali methods (a) and SILICOS-IT method (b). Red lines correspond to the fitting of data, linear fit for the values obtained using ESOL and Ali methods (a) and polynomial fit of order 3 for the values obtained using SILICOS-IT method (b).

PLA is a hydrophobic polymer, its aqueous solubility decreasing as its molecular weight increases [5]. Within this study, we have assessed the dependence of the logarithm of the molar solubility in water (log *S*) of OLAs on the molecular weight and log *P*, respectively. The SwissADME tool allowed to compute the values of the logS using the three methods described above. The obtained logS values are plotted against the molecular weight (Figures 2) and log *P* (Figures 3) and they were fitted with appropriate curves such as to obtain the highest values of the coefficients of determination (R squared). The equations obtained by data analysis are presented in Table 2. For values computed using both ESOL and Ali methods, there is a linear decrease in the logS values of OLAs with the MW and logP respectively, the decrease described by Ali method being more pronounced. The SILICOS-IT method conducts to a polynomial fit of order 3 for the variation of the logS with *M*_W_ and log *P*, respectively (Table 2).

Excepting the lactic acid, no experimental data on the molar solubility of the other OLAs were available for comparison and for evaluating these mathematical models. Taking into account that the aqueous solubility of PLA decreases with increasing molecular weight [5] and the *R*^2^ values in Table 2, Ali method seems to be appropriate to be used to predict aqueous solubility for OLAs. The discrepancy of the three prediction may come from the data sets used to derive the models for predicting the solubility values. The data set used to derive the ESOL model (2874 compounds, predictive ability r^2^ =0.72) [15] is much larger than the data set (1265 compounds, predictive ability r^2^ = 0.869) being used in Ali model. The Ali model uses experimentally determined logP values and ESOL model uses calculated logP values (clogP). Ali and his co-worker tested the ESOL model for 489 compounds with measured values for logP, solubility and melting points, and there was an improvement of the model [16]. Furthermore, Ali method explicitly accounts for the effect of polar and polarizable atoms on aqueous solubility. The discrepancy of the prediction models also underlines the limitations of the *in silico* studies and the necessity of further experimental measurements such as to improve our understanding of OLAs solubility.

We have also assessed the ADMET properties of short OLAs. Data obtained using admetSAR2.0 and SwissADME computational tools are illustrated in Tables 3 and 4. Numerical data represent the values of the probabilities that OLAs have (positive values) or have not (negative values) a certain biological action. Usually, there is a good correlation between the predictions obtained using the two computational tools. Furthermore, many of the predictions that we have obtained for OLAs are in good agreement with experimental literature data concerning the biological actions of PLA: estrogenic effects [13], good gastrointestinal absorption [32], weak potential of P-gp inhibition [33, 34], binding to human serum albumin [35, 36], penetration of the blood brain barrier [37].

Data presented in Table 3 illustrate that PLA short oligomers are predicted to have good gastrointestinal absorption and they are not considered substrates of the P-glycoprotein. The good gastrointestinal absorption of PLA has been also observed by Fernandez et al (2017) [32] by modeling the PLA nanoparticles absorption under gastrointestinal conditions. Their study revealed that the intestinal absorption of grape seed and skin extracts encapsulated in PLA nanoparticles was significantly increased.

AdmetSAR tool reveals that oligomers containing between 7 (*M*_W_ = 523 Da) and 40 (*M*_W_ = 2900 Da) lactic acid units illustrate a mean probability to be inhibitors of P-glycoprotein, this probability increases with the chain length. This prediction is in good agreement with published data by Li and his co-workers (2013) [33]. The study of Li and co-workers revealed that copolymers of mPEG-PLA could inhibit P-gp mediated efflux and that concentration played a major role in the P-gp inhibition activity. Polyethylene glycol (PEG) is a known P-gp inhibitor [34] and the fact that the inhibitory effect on P-gp efflux of the copolymers mPEG-PLA having the same PEG chain length is depended on the PLA chain length, underlines that PLA also has an inhibitory effect on P-gp activity. The copolymer with PLA chain length of 4802 Da was the most efficient P-gp activity inhibitor and copolymers with longer or shorter PLA chain lengths, gradually showed weaker inhibitory potential on P-gp function [33].

There is a disparity between the predictions made by the two computational tools concerning the ability of OLAs to penetrate the blood-brain barrier, admetSAR predicts high probabilities of penetration of blood-brain barrier and SwissADME outcomes reveal that OLAs are not able to penetrate this barrier. SwissADME tool uses for computing blood-brain–barrier permeation a model containing 260 molecules (156 permeant and 104 non-permeant) with reliable measurements of blood–brain partition and has a classification accuracy of 90% [38]. admetSAR tool uses for predicting blood barrier penetration a binary model containing a higher number of compounds (1839 with 1438 permeant and 401 no-permeant), has an accuracy of prediction of 90.7% and a specificity of 86.2% [18]. The *in vivo* experiments proved that the PLA nanoparticles are able to penetrate BBB using transcytosis by microvascular endothelial cells [34]. admetSAR2.0 tool seems to better predict the ability of the blood barrier permeation for OLAs. The disagreement of the predictions made by the two computational tolls also underlines the limitations of the computational evaluation of the biological effects of chemicals and the necessity to perform experimental studies to assess this property for OLAs.

Data from Table 3 also illustrate that the probability of OLAs to bind to plasma proteins is reduced and it decreases with increasing the chain length. Earlier studies revealed that lactic acid is able to bind to bovine serum albumin [35] and adsorption/desorption of human serum albumin at the surface of PLA nanoparticles (NPs) that decreased with increasing the diameter of NPs has been observed [36].

Data presented in Table 4 illustrate that OLAs are not considered substrates and inhibitors of the human cytochromes involved in the metabolism of xenobiotics and their presence in the human organism do not interfere with other compounds that are metabolized by these enzymes. We were not able to find scientific literature mentioning the effects of OLAs or PLA on the human cytochromes.

**Figure 4.**
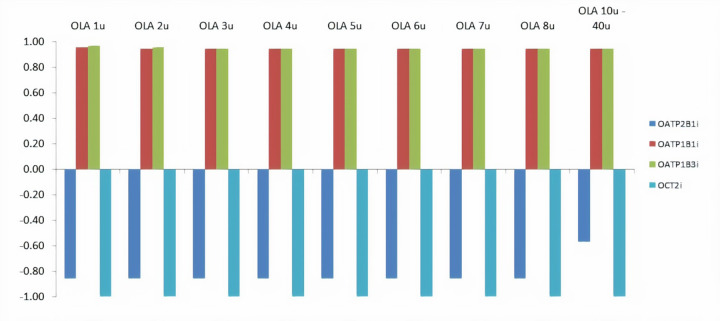
Probabilities of investigated OLAs to inhibit (positive values) or not inhibit (negative values) the organic anions and/or cations transporters (OATP/OCT) obtained using admetSAR tool. The notation ”u” refers to the number of the monomers in the oligomer.

The probabilities corresponding to the ability of investigated OLAs to inhibit the organic anions and/or cations transporters are presented in Figure 4. Figure 4 illustrates that OLAs are able to inhibit the liver-specific organic anion transporters OATP1B1 and OATP1B3. This possibility should be addressed in experimental studies as these transporters are of particular importance for hepatic pharmacokinetics and elimination of xenobiotics, and the inhibition of these transporters may result in drug–drug interactions [39].

**Figure 5.**
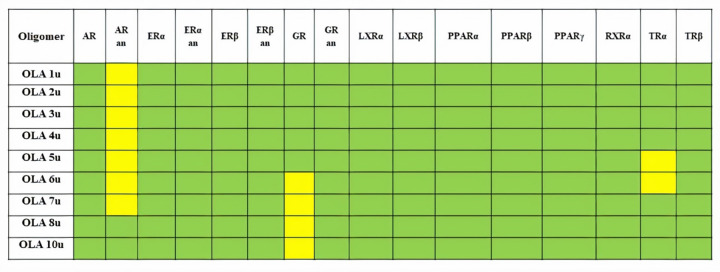
Predictions obtained using Endocrine Disruptome computational tool to assess the endocrine disruption potential of investigated OLAs: AR - androgen receptor, ERα and ERβ - oestrogen receptors α and β, GR - glucocorticoid receptor, LXRα and LRXβ - liver X receptors α and β, PPRAα, PPRAβ and PPRAγ peroxisome proliferator activated receptors α, β/δ and γ, RXRα - retinoid X receptor α, TRα and TRβ - thyroid receptors α and β. Both agonistic and antagonistic (an) effects for the nuclear receptors AR, ERα, ERβ and GR are predicted. The notation ”u” refers to the number of the monomers in the oligomer. Oligomers containing more than 10 lactic acid units were too big to accommodate in the active sites of the considered nuclear receptors and the computations have been aborted.

The potential of endocrine disruption of small oligomers of OLAs has been obtained using ENDOCRINE DISRUPTOME computational tool and is presented in Figure 5. Oligomers containing more than 10 lactic acid units were too big to accommodate in the active sites of the considered nuclear receptors and the computations have been aborted. Investigated oligomers containing up to 7 lactic acid units present a small binding capacity to the androgen receptor in the antagonistic conformation, and they may produce reproductive dysfunctions. Oligomers containing from 6 to 10 lactic acid units may affect the glucocorticoid receptor. These results are in good agreement with published data, Zimmerman et al (2019) [13] illustrated that PLA may have estrogenic effects.

**Figure 6.**
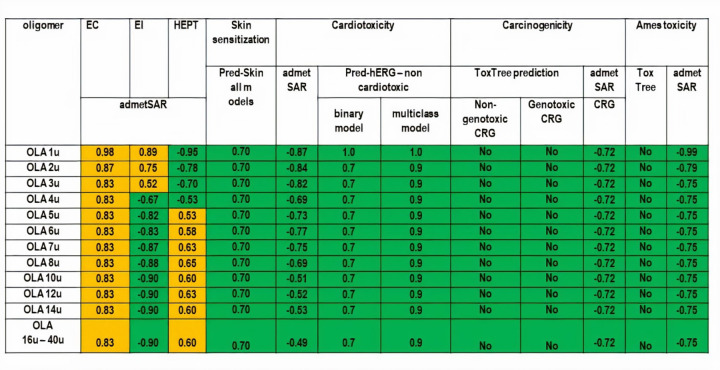
Predictions of organ and genomic toxicity of small OLAs: green cells illustrate non-toxicity and yellow cells illustrate possible toxicological effects. The notation ”u” refers to the number of the monomers in the oligomer and CRG means carcinogenicty.

The results that we have obtained for the organ and genomic toxicity of small OLAs using all considered computational tools mentioned above are presented in Figure 6. None of the investigated compounds reflects skin sensitization potential, cardiotoxicity, carcinogenicity and mutagenicity. All investigated oligomers are considered to be able to produce eye corrosion and smaller oligomers (from 1 to 3 lactic acid units) may also produce eye irritations and OLAs containing at least 5 units of lactic acids emphasize a weak potential of hepatotoxicity.

Literature data reveal one case of blindness and ophthalmoplegia following a treatment of the left periorbital region with the subcutaneous filler of PLA [40]. *In vitro* and *in vivo* animal data illustrated that lactic acid has skin and eye irritation potential (PubChem, accessed in 24 of April, 2020).

Also, literature data mention non-carcinogenicity [41], non-mutagenicity [42], non-hepatotoxicity and non-skin sensitization potential [43] of PLA. We were not able to find information concerning the cardiotoxicity of this polymer and of the investigated oligomers.

## Conclusions

Within this study, we have predicted the solubility and ADMET profiles of short oligomers of poly-lactic acid (containing from 1 to 40 lactic acid units, OLAs) that may be released during the degradation in the human organisms. The outcomes of our computational study reveal a linear decrease of the solubility of OLAs with molecular weight and logP respectively. The low aqueous solubility of OALs is an important factor limiting the hydrolysis processes and the linear decreases of logS with the molecular weight may be used as an evaluating tool for predicting the OLAs behaviour in aqueous environments.

Although the oligomers of the biodegradable polymers are considered to be easily excreted through common metabolic pathways, their possible toxic effects need to be deeply understood and detailed studies must be conducted. ADMET profile assessment for investigated OLAs reveals their favourable pharmacological profiles and limited toxicological effects on humans. According to the outcomes of the computational tools that we have used, these oligomers may inhibit the organic anion transporting peptides OATP1B1 and/or OATP1B3, they illustrate a minor probability of affecting the androgen and glucocorticoid receptors, have a weak potential of hepatotoxicity, and may produce eye injuries.

These outcomes may be used to define further experimental measurements needed to improve our understanding of the effects of the product of PLA degradation.

## Figures and Tables

**Table 1.** Short presentation of the computational tools that were used in the present study. QSAR – Quantitative Structure-Activity Relationship

Tool	Method	Output	Accuracy of predictions, %	References
SwissADME	expert-rules based 2D QSAR	Drug likeness, pharmacokinetic profile specifying Yes or NO for every investigated biological action	72-94	[14]
admetSAR2.0	2D QSAR	Pharmacokinetic profiles, organ (eye, heart, liver) and genomic toxicity specifying the probability of the presence or absence of a biological action.	72-77	[18, 19]
Pred-hERG	2D QSAR	Ability of a chemical compound to inhibit the human ether-à-go-go related gene (hERG)K+ channels using both a binary and a multiclass model.	70-89	[21, 30, 31]
Pred-Skin	2D-QSAR	Skin sensitization potential based on multiple QSAR models: prediction by binary model using human data, binary and multiclass predictions of murine skin sensitization potential based on animal data, and binary predictions based on non-animal data, i.e Direct Peptide Reactivity Assay (DPRA), KeratinoSens, and the human Cell Line Activation Test (h-CLAT)]	70-84	[20, 21, 31]
Endocrine Disruptome	Molecular docking	Probability of binding to nuclear receptors.	70-90	[22]
Toxtree	expert-rules based	Carcinogenic and mutagenic potential expressed by Yes or No.	70	[23]
CarcinoPred-EL	2D QSAR	Carcinogenic potential expressed by Yes or No.	70	[24]

**Table 2.** Equations corresponding to data analysis of the molar solubility in water (log *S*) values plotted against molecular weight (*M*_W_) and partition coefficient (log *P*) respectively for the three used methods to compute log *S* values ESOL, Ali and SILICOS-IT.

Method/ Parameter	*M*_W_		Log *P*	
	equation	*R* ^2^	equation	*R* ^2^
ESOL	log *S* =0.61-0.006 *M*_W_	0.9996	log *S* =-0.82-2.51log *P*	0.9987
Ali	log *S* =0.88-0.012 *M*_W_	0.9997	log *S* =-1.92-4.89 log *P*	0.9989
SILICOS IT	log *S* =1.02-0.002 *M*_W_ + 1.15.10^-6^ *M*_W_^2^-1.8.10^-10^ *M*_W_^3^	0.9767	log *S* =0.66-0.51 log *P* + 0.17 log *P* ^2^-0.012 log *P* ^3^	0.9923

**Table 3.** ADMET properties of OLAs: GI – gastrointestinal absorption, BBBP – blood-brain barrier penetration, P-gps- substrate of the glycoprotein P, P-gpi- inhibitor of the glycoprotein P, PPB – plasma proteins binding. The numerical values in this table represent the probabilities that investigated oligomers have (positive values) or have not (negative values) a certain biological action. The notation ”u” refers to the number of the monomers in the oligomer.

Oligomer/ biological action and tool	GI		BBBP		P-gps	P-gpi		PPB
	Admet SAR	Swiss ADME	Admet SAR	Swiss ADME	Admet SAR	Admet SAR	Swiss ADME	Admet SAR
OLA 1u	0.86	high	0.95	No	-1.00	-0.99	No	0.63
OLA 2u	0.90	high	0.98	No	-0.99	-0.98	No	0.63
OLA 3u	0.90	high	0.98	No	-0.99	-0.94	No	0.58
OLA 4u	0.90	low	0.98	No	-0.99	-0.85	No	0.55
OLA 5u	0.90	low	0.98	No	-0.99	-0.66	No	0.53
OLA 6u	0.90	low	0.98	No	-0.99	-0.49	No	0.52
OLA 7u	0.90	low	0.98	No	-0.99	0.62	No	0.52
OLA 8u	0.90	low	0.98	No	-0.99	0.68	No	0.51
OLA 10u	0.90	low	0.98	No	-0.99	0.73	No	0.50
OLA 12u	0.90	low	0.98	No	-0.99	0.74	No	0.50
OLA 14u	0.90	low	0.98	No	-0.99	0.74	No	0.49
OLA 16u- OLA 40u	0.90	low	0.98	No	-0.99	0.74	No	0.49

**Table 4.** Probabilities that OLAs are substrates (s) and inhibitors (i) of the human cytochromes (CYP) involved in the metabolism of xenobiotics. The values in this table represent the probabilities that investigated oligomers are (positive values) or are not (negative values) substrates or inhibitors of cytochromes. The notation ”u” refers to the number of the monomers in the oligomer.

Tool/ Cytochrom/ Oligomer		OLA 1u	OLA 2u	OLA 3u - OLA 40u
admetSAR	CYP3A4s	-0.84	-0.71	-0.69
	CYP2C9s	0.60	0.60	0.60
	CYPSD6s	-0.87	-0.88	-0.88
	CYP3A4i	-0.98	-0.97	-0.95
	CYP2C9i	-0.88	-0.91	-0.93
	CYP2C19i	-0.98	-0.96	-0.97
	CYP2D6i	-0.98	-0.96	-0.95
	CYP1A2i	-0.97	-0.98	-0.98
Swiss ADME	CYP3A4i	No	No	No
	CYP2C9i	No	No	No
	CYP2C19i	No	No	No
	CYP2D6i	No	No	No
	CYP1A2i	No	No	No
